# A Temporal Sampling Basis for Visual Processing in Developmental Dyslexia

**DOI:** 10.3389/fnhum.2020.00213

**Published:** 2020-07-08

**Authors:** Kim Archer, Kristen Pammer, Trichur Raman Vidyasagar

**Affiliations:** ^1^Applied Psychology and Human Factors Laboratory, School of Psychology, University of Newcastle, Newcastle, NSW, Australia; ^2^Visual and Cognitive Neuroscience Laboratory, Department of Optometry and Vision Sciences, University of Melbourne, Parkville, VIC, Australia; ^3^The Florey Institute of Neuroscience and Mental Health, Parkville, VIC, Australia

**Keywords:** dyslexia, reading, temporal sampling, magnocellular, dorsal, theta, gamma, oscillations

## Abstract

Knowledge of oscillatory entrainment and its fundamental role in cognitive and behavioral processing has increasingly been applied to research in the field of reading and developmental dyslexia. Growing evidence indicates that oscillatory entrainment to theta frequency spoken language in the auditory domain, along with cross-frequency theta-gamma coupling, support phonological processing (i.e., cognitive encoding of linguistic knowledge gathered from speech) which is required for reading. This theory is called the temporal sampling framework (TSF) and can extend to developmental dyslexia, such that inadequate temporal sampling of speech-sounds in people with dyslexia results in poor theta oscillatory entrainment in the auditory domain, and thus a phonological processing deficit which hinders reading ability. We suggest that inadequate theta oscillations in the visual domain might account for the many magno-dorsal processing, oculomotor control and visual deficits seen in developmental dyslexia. We propose two possible models of a magno-dorsal visual correlate to the auditory TSF: (1) A direct correlate that involves “bottom-up” magnocellular oscillatory entrainment of the visual domain that occurs when magnocellular populations phase lock to theta frequency fixations during reading and (2) an inverse correlate whereby attending to text triggers “top-down” low gamma signals from higher-order visual processing areas, thereby organizing magnocellular populations to synchronize to a theta frequency to drive the temporal control of oculomotor movements and capturing of letter images at a higher frequency.

## Introduction

Developmental dyslexia (which will henceforth be referred to simply as “dyslexia”) involves deficits in phonological processing of written and spoken language and visual processing of text (Snowling, 1981; Stein and Fowler, 1981; Wagner and Torgesen, 1987; Treisman, 1988; Goswami and Bryant, 1990; Livingstone et al., 1991; Cornelissen et al., 1995; Stein and Walsh, 1997; Vidyasagar, 1999; Giraldo-Chica et al., 2015). Substantial evidence suggests that impaired auditory processing reflects a deficit whereby neuronal populations fail to phase-lock to the temporal frequency of speech sounds. This underlies impaired coding of spoken syllables and phonemes, which is essential to phonological processing [Goswami’s temporal sampling framework (TSF), 2011]. Studies support the notion that temporal sampling of the rhythmic aspects of spoken language (such as syllables and prosody) enhances neural synchrony specifically at theta and gamma frequencies (Goswami and Bryant, 1990; Spironelli et al., 2006, 2008; Huss et al., 2011; Hämäläinen et al., 2012; Power et al., 2012; Goswami and Leong, 2013; Goswami et al., 2014; Colling et al., 2017; Goswami, 2018). Conversely, dyslexic readers have demonstrated impaired sensitivity to speech sounds, and lower theta and gamma synchronization in the auditory domain (Leong et al., 2011; Hämäläinen et al., 2012; Goswami et al., 2014; Leong and Goswami, 2014; Hyafil et al., 2015). However, investigation into oscillatory (brainwave) activity in the visual domain in dyslexia is limited. Cortical activity in general demonstrates remarkable consistency across operational domains – a cortical parsimony, where the brain uses similar mechanisms to support different types of cognitive function and behaviors (Bressler and Menon, 2010; Wang, 2010). Such cortical parsimony might logically apply to dyslexia, where deficits in the visual and auditory domains share common neurological mechanisms. It is well established that oscillatory phase-locking to visual stimuli occurs during visual processing (Gray and Singer, 1989; Engel et al., 1991, 2001; Kruse and Eckhorn, 1996; Herrmann, 2001; Thut and Miniussi, 2009; Mathewson et al., 2012; Thut et al., 2012; de Graaf et al., 2013; Spaak et al., 2014; Notbohm et al., 2016; Kizuk and Mathewson, 2017), and typical reading has been associated with changes at the theta (Klimesch et al., 2001; Bastiaansen et al., 2005, 2008; Meyer et al., 2015; McLelland et al., 2016), gamma (Penolazzi et al., 2009; Wang et al., 2018), and combined theta–gamma frequencies (Weiss et al., 2005; Hald et al., 2006; Bastiaansen et al., 2010; Wang et al., 2012b; Molinaro et al., 2013; Bedo et al., 2014). Early evidence also indicates that dyslexic readers have altered patterns of neuronal connectivity (Žarić et al., 2017) and lower levels of theta activity compared to typical readers (Stokić et al., 2011; González et al., 2016).

A generalized cross-domain temporal sampling deficit in dyslexia has been proposed, and already has some theoretical support (Llinás, 1993; Johnston et al., 2008; Goswami, 2011; Kraus, 2012; Vidyasagar, 2013, 2019; Gori et al., 2014; Pammer, 2014; Lewis and Bastiaansen, 2015; Hancock et al., 2017; Stein, 2019). Here, we aim to expand on the evidence of oscillatory phase-locking in visual processing and theta-gamma activity during typical reading and propose a visual correlate to the auditory TSF of dyslexia. Consistent with the auditory TSF we suggest that impaired oscillations in the visual domain – specifically at the theta and gamma frequencies – hinder effective visual temporal sampling and parsing of text.

We also aim to provide a possible link between the auditory TSF and another dominant theory of dyslexia, that is the magno-dorsal deficit hypothesis. Considerable evidence indicates the visual deficits in dyslexia are associated with changes to the magnocellular pathway within the dorsal visual stream^1^ which results in difficulties encoding and processing the low-spatial high-temporal characteristics of text (Badcock and Lovegrove, 1981; Stein, 1991; Cornelissen and Hansen, 1998; Slaghuis and Ryan, 1999; Vidyasagar, 1999, 2004; Vidyasagar and Pammer, 1999; Facoetti et al., 2006). We hypothesize that this magno-dorsal deficit is one which affects neural synchrony within and between magnocellular dominated visual pathways. Generalized magnocellular abnormalities as a result of early immune attack or genetic anomalies (Cowan, 1970; Cook and Stevens, 1973; Paracchini et al., 2016; Jiménez-Bravo et al., 2017) may influence the power and phase-coordination of neural firing (Eysel and Wolfhard, 1984; Yücel et al., 2003; Jiménez-Bravo et al., 2017). This could happen in one two ways. The generation of neuronal oscillations and synchrony within clusters of neurons and/or between clusters of neurons in different areas can be affected if timing of neuronal events is disrupted. Another additional factor is the morphological pathology in an area that could alter the resonance frequency of cells in responding to top-down signals, the resonance frequency of neurons being dependent on their biophysical characteristics of the cell and the local circuitry it is embedded in Hutcheon and Yarom (2000), Vidyasagar (2013). Remarkable parallels to the auditory TSF can be found in the magno-dorsal stream in reading and dyslexia at both theta and gamma frequencies (see Table 1). Eye movements during reading are driven by magno-dorsal areas, and in non-dyslexic readers, occur at a systematic theta frequency. However, dyslexic readers have erratic patterns of eye movements during reading, resulting in non-rhythmic fixations and saccades (Rayner, 1998). This could be an indication of theta frequency, fixation related, action potentials – or an impairment of them – in early visual sensory areas during reading and dyslexia, respectively. Evidence of theta and gamma neuronal synchronization during visual processing can be found specifically along the magno-dorsal pathway. At the “bottom-end,” retinal gamma activity has been identified in spatio-temporal tasks that isolate magnocellular activity in normal readers, yet it is not apparent in dyslexia (Pammer and Wheatley, 2001; Kevan and Pammer, 2008; Gori et al., 2014). At the “top-end” of the pathway [the posterior parietal cortex (PPC)], visual attention and processing involves increased low gamma (25–45 Hz) synchronization (Saalmann et al., 2007), which has already been shown to be significantly lower in the auditory cortex in dyslexia (Lehongre et al., 2011). We suggest that theta–gamma cross-frequency coupling, which occurs when processing spoken language, also occurs along the magno-dorsal pathway during top-down attentional modulation and bottom-up processing of visual stimuli (von Stein and Sarnthein, 2000; Demiralp et al., 2007; Holz et al., 2010; Wang et al., 2010).

**TABLE 1:** Parallels in neuronal activity in the auditory temporal sampling framework and magno-dorsal hypothesis of reading and dyslexia.

	Theta activity	Gamma activity
Auditory temporal sampling framework	• Phase-locking to theta frequency syllabic sounds, impaired in dyslexia	• Phase-locking to gamma frequency phonemic sounds, impaired in dyslexia
		• Cross-frequency coupling to enable higher-order phonological processing, impaired in dyslexia

Magnocellular-dorsal network	• Theta frequency eye-movements during non-dyslexic reading; absence of theta rhythm eye-movements in dyslexic readers	• FD illusion demonstrates higher threshold (impaired) in dyslexia to high frequency temporal + magnocellular- sensitive task compared to typical readers
	• Projections form magno-dorsal areas (PPC and MT/V5) involved in oculomotor control during reading	• Gamma in posterior parietal cortex enables higher-order visual processing, significantly lower in dyslexia
		• Top-down gamma inducement with visual attention
	• Claustral theta output might promote neural synchrony between dorsal and early visual cortical areas	• Gamma activity in LGN and visual cortex

After reviewing the literature on the current neurobiological theories of reading and dyslexia and the evidence for them, we will consider the possibility of a visual TSF, where theta and gamma activity along the magno-dorsal pathway enable the visual cognitive processes required for reading. We will present two alternative models of a magno-dorsal TSF. Model 1 is a direct correlate to the auditory TSF, whereby theta frequency eye movements act as a stimulus that causes oscillations in visual sensory areas to synchronize at theta, and thereby induce higher-order magno-dorsal gamma activity. A potential flaw with this model is that, unlike listening to spoken language (an external environmental stimuli), attending to text is a process driven internally by the reader. Considering this, we also present Model 2, a top-down-bottom-up magno-dorsal oscillatory circuit where endogenous gamma synchronization enables top-down direction of visual attention to text, and couples with theta signals in early visual sensory areas for the bottom-up temporal filtering of text.

## Cognitive Deficits in Developmental Dyslexia

Dyslexia is a universal and persistent form of low literacy in which a child fails to learn to read at an age-appropriate rate (e.g., Snowling, 2019). The disorder is found across languages and is not associated with intelligence, age, education or other neurological deficits (Snowling, 2019). It is well recognized that dyslexia is associated with cognitive deficits in auditory processing of spoken language and visual processing of text – both of which may be essential for a child to learn to read.

Auditory processing of spoken language is fundamental for the development of phonological and lexical knowledge. Phonological awareness in children is a strong predictor of reading ability later in life (Whalley and Hansen, 2006), and it is well recognized that this is impaired in most children with dyslexia (Goswami and Bryant, 1990; Snowling, 2001; Ziegler and Goswami, 2005; Goswami, 2011; Leong and Goswami, 2014), even though a clear causal connection has not been established (Castles and Coltheart, 2004; Vidyasagar and Pammer, 2010; Vidyasagar, 2012). In typical readers, phonological knowledge is amassed via decoding sound components (phonemes) in spoken language (Spiro and Myers, 2002). This requires recognizing the temporal order of speech sounds, rapid and accurate word recognition, rhythmic stress, and coding information in short-term memory (Hunt et al., 1975; Goswami and Leong, 2013). Decreased perceptual sensitivity to sound-segments in language, rhythmic stress, and timing of speech have been demonstrated in dyslexia, indicating problems in auditory processing (Goswami and Bryant, 1990; Snowling, 2001; Ziegler and Goswami, 2005; Goswami and Leong, 2013; Goswami et al., 2013).

During fluent reading, phonological knowledge is coordinated with visual processing to make lexical sense of images captured as the eye moves along a passage of text (Heth and Lavidor, 2015). This requires effective oculomotor control and rapid higher-order visual processing, both of which are disrupted in dyslexia. Dyslexic readers have difficulties conducting visual search and orienting attention among cluttered environments, (Vidyasagar and Pammer, 1999, 2010; Facoetti et al., 2006, 2010; Sireteanu et al., 2008; Moore and Schwitzgebel, 2018), processing low spatial frequency arrays (Badcock and Lovegrove, 1981; Slaghuis and Ryan, 1999), feature binding (Pammer et al., 2004a), coding the temporal sequences of words and letters within words (Stein, 1991; Cornelissen and Hansen, 1998; Vidyasagar, 1999, 2004; Vidyasagar and Pammer, 1999; Facoetti et al., 2006), and convergence of images from left and right eyes (Jainta and Kapoula, 2011).

**FIGURE 1:**
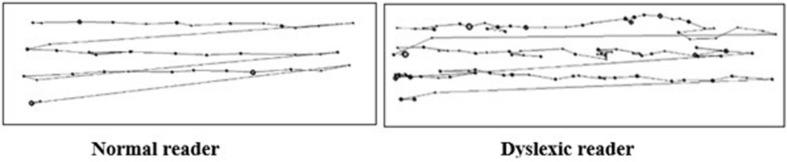
Comparison of eye-movements during reading. **(Left image)**: Normal reader. Fixations (represented by dots) and saccades (represented by the connecting lines) occur in a linear fashion at an approximate temporal frequency of 4 Hz. **(Right image)**: Dyslexic reader. Fixations (represented by dots) occur erratically with no rhythmic temporal pattern (figure adapted from Prado et al., 2007).

Dyslexic children also display differences in oculomotor control during reading. Typical readers move their eyes linearly and rhythmically across text, making continuous saccades and fixations (Rayner, 1998). Saccades move the eye from one location to the next over a period of approximately 25–50 ms. Their purpose is to bring a new region of text into foveal vision – the area of the eye that provides the highest visual resolution. Between saccades, the eye remains relatively still for 200–300 ms in a fixation, allowing for more detailed analysis of a section of text (Rayner, 1998). Predominately, the eye progresses forward (left-to-right in left-to-right scripts or the reverse in right-to-left scripts) along a line of text making regular progressive fixations. Occasionally, the eye moves backward and fixates on a section of text that has already been read. This is known as regressive fixation and occurs when text needs revising for comprehension (Rayner, 1998). These eye movements differ significantly in dyslexia. Children with dyslexia also do not demonstrate normal developmental gains in eye movements when reading (see Figure 1). Typically, as children gain proficiency in cognitive processing during reading, they are able to move their eyes more rapidly along a passage of text. Progressive fixations become shorter, saccades travel further and fewer regressive fixations are required (Rayner, 1998). However, these oculomotor developmental gains are not evident to a similar extent in dyslexic children (Lefton et al., 1979; Stein, 1991; Benfatto et al., 2016). Child and adult dyslexic readers demonstrate erratic visual search patterns and make more frequent, lengthier fixations in comparison to non-dyslexic readers (Adler-Grinberg and Stark, 1978; Eden et al., 1994; Lefton et al., 1979; Martos and Vila, 1990; Stein, 1991). As such, dyslexic readers do not demonstrate an efficient rhythmic pattern of saccades and fixations.

## Neurophysiological Theories and Evidence Pertinent to Reading and Dyslexia

### The Auditory Temporal Sampling Framework

The neurobiological aetiology of impaired language processing is thought to be a deficit in the synchronization of oscillatory populations in the auditory domain, whereby coding of spoken language is impaired due to inept neuronal phase-locking to the theta temporal frequency of spoken language. It is well established that neuronal populations actively react to rhythmic events in the environment (Buzsáki and Draguhn, 2004; Lakatos et al., 2008; Calderone et al., 2014). When neuronal populations in sensory brain areas attend to repetitive stimuli they phase-lock (“tune in”) to the temporal structure of that stimulus and synchronize their firing rate to that frequency, a process called oscillatory entrainment (Lakatos et al., 2008; Calderone et al., 2014). Temporal sampling and oscillatory entrainment at low frequencies are thought to be crucial to the perceptual sensory processing, allowing environmental stimuli with repetitive and predictable rhythms – such as speech – to be distinguished more easily than unpredictable non-rhythmic stimuli (Buzsáki and Draguhn, 2004; Luo and Poeppel, 2007; Schroeder and Lakatos, 2009; Calderone et al., 2014).

**FIGURE 2:**
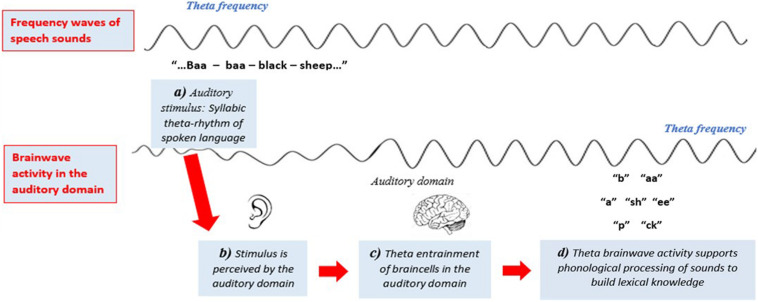
Stylized representation of Goswami’s (2011) auditory temporal sampling framework (TSF). Syllabic sounds in spoken language occur at a theta frequency. It is hypothesized that when neuronal populations in the auditory domain attend to speech-sounds they phase-lock (entrain) to this theta frequency. Figure adapted from Pammer (2014).

The auditory temporal sampling hypothesis builds on the understanding that the regular rhythmic sequence of spoken syllabic sound-segments occurs at an approximate theta frequency (Goswami, 2011). That is, spoken syllables occur approximately every 200–300 ms. It is thought that neuronal populations in the auditory domain phase-lock to speech-sounds, synchronizing their oscillations to a theta frequency and initiating the cortical activity required for cognitive encoding of amplitude and temporal order information of phonemes (Goswami and Bryant, 1990; Luo and Poeppel, 2007; Spironelli et al., 2006, 2008; Goswami, 2011, 2018; Hämäläinen et al., 2012; Goswami and Leong, 2013; Goswami et al., 2014). It is thought that unstable theta phase-locking in sensory auditory areas results from an oscillatory synchronization deficit throughout the auditory pathway, disrupting effective phonological processing (Goswami, 2011) (refer to Figure 2). Compared to typical readers, children with dyslexia have significantly reduced theta activity when completing phonological tasks (Spironelli et al., 2006, 2008) and when listening to theta frequency auditory stimuli (Huss et al., 2011; Hämäläinen et al., 2012; Power et al., 2012; Colling et al., 2017). Decreased perception to speech-sound frequencies have also been identified in newborns at risk of dyslexia (Thiede et al., 2019). However, low-frequency theta waves in sensory areas alone are insufficient for enabling higher temporal rates of parsing needed for complex cognitive processing of language. It is thought that higher-order phonological processing is enabled by cross-frequency coupling of phase-locked theta oscillations to gamma oscillations (Lehongre et al., 2011, 2013; Gross et al., 2013; Lizarazu et al., 2015).

#### Cross-Frequency Oscillatory Coupling

Cross-frequency coupling is a well-recognized mechanism by which an oscillatory population synchronized at one frequency modulates another neuronal population to synchronize at a distinct, second, frequency (Canolty et al., 2006; see Jensen and Colgin, 2007 for a review). Coupling of theta and gamma oscillations in particular occurs throughout the brain, and has been demonstrated in the hippocampus (Bragin et al., 1995; Axmacher et al., 2010; Belluscio et al., 2012), auditory cortex (Lakatos et al., 2007; Fontolan et al., 2014), posterior parietal cortex (Hawellek et al., 2016), and between the frontal and posterior parietal cortices (Friese et al., 2013). Coupling of alpha and gamma frequencies has also been shown between parietal and frontal areas (Wang et al., 2012a). Functionally, cross-frequency coupling leads to modulation of high frequency oscillations by low frequency oscillations, enabling entrainment to behaviorally relevant external sensory occurrences and internal cognitive events (Canolty and Knight, 2010). It is also an important mechanism for rapid neuronal communication between distant brain locations (Schroeder and Lakatos, 2009), with gamma oscillations operating as a functional mechanism at the local population level, and theta oscillations working to temporally coordinate activities of these populations across large-scale cortical networks (Lisman and Buzsaki, 2008).

Theta–gamma cross-frequency coupling is thought to be integral to speech encoding (Hyafil et al., 2011, 2015; Gross et al., 2013). After entraining to the syllabic structure of speech sounds, lower-order theta oscillations drive synchronized neuronal spiking at gamma frequencies (such that gamma activity becomes nested within synchronized theta spikes) to enable higher-order auditory encoding (Lakatos et al., 2005; Hyafil et al., 2011, 2015). When processing speech, theta-gamma coupling could enhance signal transfer, as well as increasing the amplitude of gamma waves to enable swift higher-order phonological decoding of speech (Lakatos et al., 2005; Hyafil et al., 2011, 2015; Gross et al., 2013).

Another possibility that underlies speech encoding is that coupled theta and gamma oscillations are in play throughout the auditory network in a bottom-up response to two distinct temporal frequencies in speech (Ghitza, 2011; Giraud and Poeppel, 2012). Generally, a theta–gamma “neural code” is thought to enable discrimination and temporal ordering of sequential sensory stimuli (Lisman and Buzsaki, 2008). As well as the theta frequency of syllabic chunks (approximately four syllables per second), speech acoustics simultaneously consist of high gamma frequency phonemes (the smallest constituent of language) (Ghitza, 2011; Giraud and Poeppel, 2012). In order to process syllables and phonemes, the neuronal response to speech might be temporal sampling at theta and gamma frequencies simultaneously (Ghitza, 2011; Giraud and Poeppel, 2012). Theta and gamma frequency oscillations might harmonize in a bottom-up manner for early encoding of both aspects of the speech acoustic. This model of theta–gamma activity has indeed been demonstrated whereby each frequency not only tracks the syllabic and phonetic components of speech respectively, but coordination of theta–gamma oscillations enables the listener to code the temporal order of phonemes within a syllable (Hyafil et al., 2015).

Thus, cross-frequency coupling enables sensory areas of the brain which capture language stimuli to communicate rapidly with higher-order brain areas for real-time processing of language input (Ghitza, 2011; Giraud and Poeppel, 2012; Hyafil et al., 2015). We propose that it is also possible that theta–gamma cross-frequency coupling occurs not only during auditory processing of spoken language, but also during visual processing of written text (von Stein and Sarnthein, 2000; Demiralp et al., 2007; Holz et al., 2010; Wang, 2010; Vidyasagar, 2013), and that such theta–gamma coupling may be impaired in the visual as well as auditory domains in dyslexia (Vidyasagar, 2013).

### Theta and Gamma Oscillations During Reading

Investigations into cortical oscillatory activity during typical reading have found changes in gamma, theta, and combined gamma–theta activity during word and sentence processing. Significant increases in widespread cortical theta synchronization have been observed during reading (Klimesch et al., 2001), as well as localized theta increases over the occipital cortex (Bastiaansen et al., 2008), frontal cortex (Meyer et al., 2015), and posterior cortex (Meyer et al., 2015). These studies used word recognition and lexical-semantic violation tasks, leading to conclusions that theta synchronization enables working memory during reading (Meyer et al., 2015), for example lexical-semantic retrieval and word recognition (Klimesch et al., 2001; Bastiaansen et al., 2005, 2008), with the rationale that (i) temporal sequences are encoded within theta band oscillations (Lisman and Idiart, 1995; Lisman and Buzsaki, 2008) and (ii) theta waves play a key role in the maintenance of temporal order in working memory (Klimesch et al., 2001; Bastiaansen et al., 2005, 2008; Roberts et al., 2013). Remarkably, theta synchronization has been time-locked to the onset of sentence reading (Bastiaansen et al., 2010), akin to the theta phase-locking that occurs when listening to spoken language (Goswami and Bryant, 1990; Spironelli et al., 2006, 2008; Luo and Poeppel, 2007; Goswami, 2011, 2018; Hämäläinen et al., 2012; Goswami and Leong, 2013; Goswami et al., 2014).

Gamma activity has been observed during sentence reading with semantic violation tasks (Penolazzi et al., 2009) and language prediction tasks (Wang et al., 2018), leading to the suggestion that gamma frequency oscillations enable top-down linguistic predictions to pair with bottom-up input (Lewis and Bastiaansen, 2015; Wang et al., 2018). Studies have also identified increases in *both* theta and gamma oscillations during sentence reading (Weiss et al., 2005; Wang et al., 2012b; Mellem et al., 2013) and semantic processing tasks (Hald et al., 2006; Bastiaansen et al., 2010). Word-reading paradigms have demonstrated widespread (Bedo et al., 2014) and frontal-occipital (Molinaro et al., 2013) bi-directional theta and gamma phase-locking, indicating oscillatory activity is involved in both top-down attentional and predictive processes, as well as bottom-up analysis of incoming text stimuli (Molinaro et al., 2013).

Despite the above studies showing specific oscillatory dynamics in the visual areas associated with reading, there are only a few studies that have investigated oscillatory activity during reading in people with dyslexia. However, the evidence so far indicates that people with dyslexia have significantly fewer and altered neural network connections (Žarić et al., 2017), as well as lower levels of theta activity during reading (Stokić et al., 2011; González et al., 2016). Analysis of theta frequency network connections over the temporal and parietal cortices during reading indicate non-dyslexic readers have widespread oscillatory networks that are both quantitatively and qualitatively greater than those in dyslexic readers (Stokić et al., 2011). Furthermore, there is a positive correlation between reading ability and the number and power of neuronal networks in children with dyslexia (Žarić et al., 2017). Not only do connectivity networks differ during reading, EEG measures at resting-state with eyes closed show widespread theta activity is significantly less in dyslexic children compared to non-dyslexic children (González et al., 2016).

We propose that this increased theta and gamma activity during typical reading, as well as altered neuronal networks in dyslexia, are indicative of oscillatory activity in reading and dyslexia beyond just phonological processing in the auditory domain. It is possible that theta and gamma networks are involved in visual processing during reading in a manner similar to the auditory temporal sampling hypothesis (TSH). A visual TSF might also provide a theoretical link between the auditory TSF and another current influential theory of dyslexia, namely the magno-dorsal deficit hypothesis.

### Magno-Dorsal Deficit Hypothesis of Dyslexia

At present, the prominent theory accounting for visual deficits in dyslexia is an impairment in the magnocellular-dominated dorsal pathway (see Stein, 2001, 2019 for reviews). The unique physical and functional characteristics of magnocells enable visual processes essential to reading (Lovegrove, 1996). Magnocellular axons are thick and myelinated, allowing for rapid signal conduction (i.e., high-temporal frequency processing). This enables magnocells to respond quickly to stimulation and provide rapid bottom-up information transfer for the visuo-spatial attention network essential for reading (Vidyasagar, 1999; Vidyasagar and Pammer, 1999).

Magno-dorsal processing can be examined using visual paradigms which exploit magno-dorsal characteristics (see Stein, 2001 for a full review) and compared to controls, dyslexic readers have significant difficulties in magno-dorsal isolating paradigms (typically involving low-spatial – high-temporal frequency stimuli). Dyslexic readers demonstrate destabilization of the binocular fixation necessary for convergence of images from the left and right eyes (Stein and Walsh, 1997) – suggesting a possible deficit in magnocellular activity. Hindrance of saccadic suppression results in the superimposition of successive images (Stein and Walsh, 1997) and may indicate a functional deficit in MT/V5 in dyslexia. Alterations in oculomotor control and motion perception (Eden et al., 1996; Demb et al., 1998) also indicate variances in MT/V5 and/or PPC functionality and can underlie poor orthographic skills. Higher-order dorsal processing deficits in dyslexia include difficulties in orienting and allocating visual attention (Facoetti et al., 2006, 2010), sub-lexical processing (Stein, 1991; Cornelissen and Hansen, 1998; Vidyasagar, 1999, 2004; Vidyasagar and Pammer, 1999; Facoetti et al., 2006), spatial processing (Badcock and Lovegrove, 1981; Slaghuis and Ryan, 1999), and feature binding (Pammer et al., 2004a).

Deficits in magnocellular processing have been identified as early as the retina using the spatial frequency doubling (FD) illusion (Pammer and Wheatley, 2001; Gori et al., 2014). This finding is significant in that it shows differences between dyslexic and non-dyslexic readers not only in magnocellular processing *per se*, but also in oscillatory synchronization at a gamma frequency at the earliest point of the magno-dorsal pathway. The illusion is produced by a 0.1-4 c/deg grating, flickering in temporal counterphase at >15Hz (i.e., extending to the gamma frequency range), which yields the perception that the grating is double the actual spatial frequency (Kelly, 1966, 1981; Maddess and Henry, 1990). The illusion occurs due to functional characteristics of M(y) cells in the retina (Benardete et al., 1992) and lateral geniculate nucleus (LGN; Marrocco et al., 1982), akin to full-wave rectification in signal processing (Kelly, 1981). Dyslexic readers have significantly higher spatial and temporal threshold to the FD illusion presented at 18 Hz (Gori et al., 2014), 25 Hz (Pammer and Wheatley, 2001), and 50 Hz (Kevan and Pammer, 2008) compared to non-dyslexic readers, indicating that a specific magnocellular oscillatory synchronization deficit may exist as early as the retina. Pre-readers at risk of dyslexia show the same threshold differences to the FD illusion presented at 50 Hz (Kevan and Pammer, 2008) and illiterate, semi-literate, and literate adults are all equally sensitive to the illusion presented at 50 Hz (Flint and Pammer, 2011). This suggests that sensitivity to the FD illusion is not dependant on magnocellular development as a child learns to read and is therefore not a *consequence* of failing to learn to read (Kevan and Pammer, 2008; Flint and Pammer, 2019). Furthermore, individual differences in sensitivity to the FD illusion are predictive of reading performance in dyslexic children (Gori et al., 2014).

That dyslexic readers are less sensitive to the FD illusion is important in linking deficits in retinal magnocellular populations to putative deficits in neural responses to stimuli that occur at the gamma frequency at the commencement of the magno-dorsal pathway. Building on this, as well as the evidence of theta and gamma activity during reading and decreased neural connectivity in dyslexia, the remainder of this paper will explore the possibility that the visual deficits in dyslexia may be due to inadequate oscillatory synchrony within the visual system (Johnston et al., 2008; Vidyasagar, 2013). We hypothesize that unstable phase-locking at the frequencies necessary for the ordered sampling and processing of text – especially between magnocellular populations and in their gating function on other pathways – may occur in the visual domain in a manner similar to the auditory Temporal Sampling Framework or TSF (Goswami, 2011).

## Temporal Organization of Magnocells

We suggest that the deficit in dyslexia may be one of impaired functional connectivity between magnocellular-driven populations in the dorsal pathway. Damage to the neuronal structure or connections of magnocells might hinder synaptic communication within and between magnocellular-driven populations in the dorsal stream and also in their feedback signals to early visual areas, destabilizing oscillatory synchrony at the frequencies required for visual processing during reading.

The physical and functional characteristics of magnocells suggest magnocellular populations are ideal for rapid neuronal entrainment. Healthy magnocells are large and have thick, myelinated axons, which enable rapid signal communication of information. Their transient response characteristics (Dreher et al., 1976; Kaplan and Shapley, 1982; Hicks et al., 1983) make them ideally suited for achieving a high degree of precision in any kind of high frequency temporal sampling and in coincidence detection of neural spiking events, which are essential for creating a neural code based on synchronized oscillations. Thus, retinal and LGN magnocells have an inherent capacity for population-level phase-locking to repeated stimuli as their action potentials reset in phase with rapid stimulus shifts (Reinagel and Reid, 2000; Reich et al., 2001; Butts et al., 2011). By synchronizing their firing phases, magnocells dramatically increase their functional capacity and can encode larger volumes of text at a more detailed level (Destexhe et al., 1999; Agarwal and Sarma, 2012).

However, magnocells are highly vulnerable to damage – possibly from immunological attack *in utero* or genetic mutations (Cowan, 1970; Cook and Stevens, 1973; Paracchini et al., 2016; Jiménez-Bravo et al., 2017). Myelin damage has been found in dyslexic adults at autopsy (Humphreys et al., 1990), which is known to occur in response to immune attacks before neuronal myelination is complete, i.e., *in utero* or within the first year of life (Myers, 1969; Larroche, 1986). Immunological attack is also associated with neurons that are smaller in size and number (Cowan, 1970; Cook and Stevens, 1973), changes which correspond to a decline in neuronal function and transsynaptic communication (Eysel and Wolfhard, 1984; Yücel et al., 2003). Compared to controls, dyslexic readers have also been found to have fewer and smaller magnocells at autopsy, with magno-dorsal layers of the LGN being over 20% smaller than non-dyslexic readers (Livingstone et al., 1991; Galaburda et al., 1994; Fawcett et al., 1996). These alterations in cell morphology are likely to affect the functional capabilities of magnocells. Injury to myelin, impaired axonal development and dendritic arborization, as well as poor neuronal migration, would likely result in smaller, disorganized magnocellular populations which are impaired in both firing power and ability to synchronize their oscillatory phases at both the single-cell and population levels. Indeed, a number of genetic mutations in dyslexia have been identified to directly impact neuronal connectivity and have been linked to rhythmic dysfunction (see Paracchini et al., 2016; Jiménez-Bravo et al., 2017 for more detailed discussions on dyslexia and genetics).

Alongside the evidence of theta and gamma activity during reading, altered neural connections in dyslexia and retinal magnocellular temporal response functions as deduced from FD illusion studies, these genetic and physiological changes raise questions as to whether the impaired magno-dorsal processing in dyslexia may be due to impaired temporal processing in magnocellular-driven cortical circuitry. Given that impaired oscillatory synchrony occurs at theta and gamma frequencies in dyslexia in the auditory domain, cortical parsimony indicates oscillations in the visual domain in the magno-dorsal stream could also be impaired at gamma and theta frequencies, as well as affecting cross-frequency coupling of theta and gamma oscillations.

## Impaired Theta Oscillatory Synchronization in Visual Processing During Reading

**FIGURE 3:**
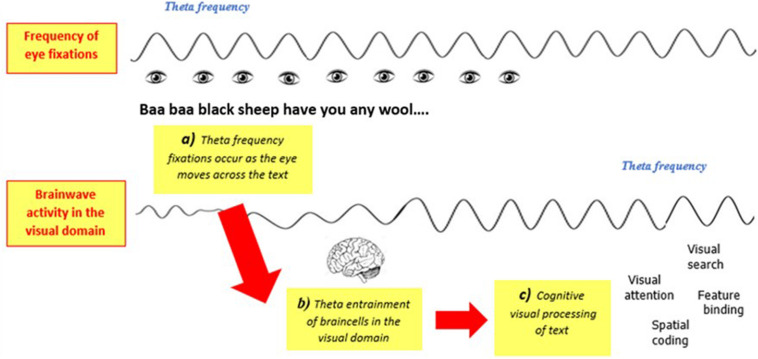
Stylized representation of a possible visual correlate to the auditory temporal sampling framework. It is hypothesized that in normal reading theta frequency fixations entrain theta brainwave activity in the visual domain, thus supporting cognitive visual processing during reading (figure adapted from Pammer, 2014).

As discussed earlier, reading entails rapid shifts in visual stimuli that occur as the eye moves along a line of text (Rayner, 1998). Given that magnocells respond to alterations in stimuli by resetting their action potentials (Reinagel and Reid, 2000; Reich et al., 2001; Butts et al., 2011), each eye movement during reading might cause a phase-reset of retinal magnocells (Marrocco et al., 1982). Lateral geniculate nucleus single cell action potentials also consistently correlate with the temporal rate of visual stimulus presentation in animal studies, which is thought to enable coding of stimulus features (Creutzfeldt and Kuhnt, 1973; Kepecs and Lisman, 2003; Samengo et al., 2013). Eye movements during reading occur systematically and rhythmically, and as highlighted by Pammer (2014) and Vidyasagar (2013) occur at low theta frequencies. During reading, the average saccade takes roughly 30 ms (Rayner, 1998) and the average fixation lasts approximately 225 ms (Rayner, 1998). Thus, during reading a new fixation commences approximately every 255 ms, equating to a temporal sampling rate of approximately 4 Hz. We propose that this is a visual correlate to the temporal sampling of theta frequency syllabic speech-sounds hypothesized in the auditory TSF (refer to Figure 3). Theta frequency eye movements may be indicative of theta frequency fixation related potentials in lower-order areas of the visual pathway, correlating to the auditory domain’s theta phase-locking in response to spoken language. Indeed, visuospatial processing has been shown to correlate with ongoing theta activity around the time of saccades (McLelland et al., 2016), and a significant increase in theta synchronization has been time-locked to onset of sentence reading (Bastiaansen et al., 2010). Furthermore, given dyslexic readers have erratic, non-rhythmic eye movements during reading, we suggest that this is indicative of unstable theta activity early in the magno-dorsal pathway. Dyslexic readers typically have a decreased temporal frequency rate of fixations due to increased fixation frequency and increased fixation dwell time (Rayner, 1998), which could be inadequate for encoding visual information.

**FIGURE 4:**
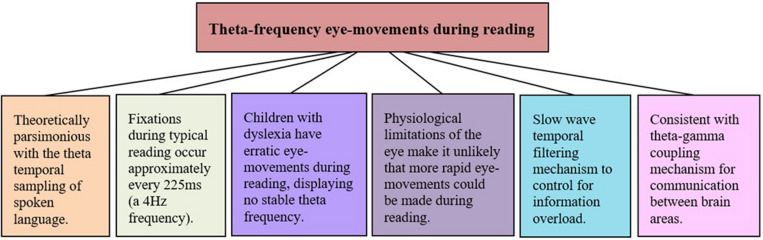
Summary of the rationales for theta-frequency eye-movements during reading, which may be orchestrated by low-frequency claustral oscillatory output.

Beyond cortical parsimony to temporal sampling of spoken language, theta frequency sampling of text makes sense both behaviorally and functionally. From a basic mechanical point of view, physiological limitations make it unlikely that systematic eye movements could occur at frequencies beyond theta. Cognitively, oscillations are thought to act as a mechanism for controlling the timing in serial processing of sensory input (Lisman and Idiart, 1995) and low frequency temporal sampling would act as a control mechanism for information overload. When encoding spoken language, theta frequency phase-locking acts as a temporal filter or funnel, whereby auditory information is segmented into chunks for systematic analysis (Stevens, 2002; Poeppel, 2003). This is because the volume capacity of neurons to encode information is bound by phase-locking to temporal changes in stimuli consistent with their temporal modulation transfer functions (Hubel and Wiesel, 1961; Hicks et al., 1983; Reinagel and Reid, 2000; Levine and Cleland, 2001; Nirenberg et al., 2001; Rathbun et al., 2010). Theta temporal sampling of text could act as a similar pre-processing or parsing of visual language. Text environments are cluttered with a low-spatial frequency between complex visual features of letters and words. The visual system could easily be overloaded and unable to effectively encode visual features if the eye were to shift too quickly along a passage of text. Low frequency temporal sampling and phase-locking could therefore act as a filter similar to the parsing of spoken language, maintaining a manageable signal-to-noise ratio of visual information (refer to Figure 4).

## Gamma Synchronization Along the Magno-Dorsal Pathway

Low-level theta encoding is clearly important in sampling text as it reflects the oculomotor rhythm at which the eye moves across the page and is disrupted in dyslexia by poorly controlled saccadic movements. However, just as theta synchronization in the auditory domain is insufficient for complex phonological processing of spoken language, theta synchronization in the magno-dorsal pathway is unlikely to be sufficient for higher-order cognitive processing of text. Indeed, we will present evidence of gamma activity that has been identified along the magno-dorsal pathway and is associated with visual information processing and attention (Herrmann and Mecklinger, 2000, 2001; Fries et al., 2002; Saalmann et al., 2007; Lehongre et al., 2011; Bastos et al., 2014). It is possible that magnocellular theta oscillations couple to gamma activity along the magno-dorsal pathway, mirroring the theta-gamma coupling that is hypothesized in the auditory TSF (von Stein and Sarnthein, 2000; Demiralp et al., 2007; Holz et al., 2010; Wang et al., 2010).

Building on the evidence discussed we will now present two possible models for a magno-dorsal visual TSF in reading and dyslexia. We will firstly consider the possibility of a direct visual correlate to the auditory TSF, where eye movements act as a stimulus to entrain theta oscillations in visual sensory areas, and couple to gamma oscillations along the magno-dorsal in a bottom-up manner (Model 1). We will then present an alternative hypothesis of a magno-dorsal TSF that is a top-down and bottom-up oscillatory circuit, where modulation of oscillations with reading initiates with gamma synchronization in the PPC to drive theta oscillations and theta frequency eye movements during reading (Model 2).

## Model 1: a Visual-Temporal Sampling Model as a Bottom-Up Process

Analogous to the packets of auditory stimuli in spoken language, the actual oculomotor mechanics of reading might entrain magnocellular theta oscillations in the visual domain (Pammer, 2014). Fixation-related potentials that occur as the eye moves along the text might account for the increase in theta power that occurs at the onset of sentence reading (Bastiaansen et al., 2010). Just as the auditory domain phase-locks to the theta frequency of syllabic speech sounds to enable phonological processing, oscillatory activity early in the visual pathway might phase-lock to theta frequency fixations that occur during reading (Pammer, 2014). Support for this can be found in the LGN, where phase-locked oscillations encode information about stimulus shape (Gawne et al., 1991; McClurkin et al., 1991), indicating the LGN uses temporal coding to maximize efficiency of information coding (Dong and Atick, 1995; Funke and Wörgötter, 1997; Reinagel and Reid, 2000; Reich et al., 2001; Butts et al., 2011). It has been argued that saccades and the eye drifts between saccades favor the processing of low and high spatial frequencies respectively (Boi et al., 2017). This means, that when applied to the effect of eye movements during reading, saccades may favor the processing of words as blurred images, but during fixations, which are associated with slow eye drifts, the finer frequencies needed for letter identification are preferentially processed. As dyslexic readers have erratic patterns of fixations and saccades during reading (Stein, 1991; Sireteanu et al., 2008), it is therefore likely they would have no stable temporal low-frequency stimulus to which magnocellular oscillations can phase-lock during reading (Pammer, 2014), accounting for poor encoding of complex low-spatial text. Interestingly, re-training eye movements in dyslexic children using low theta frequency temporal stimuli has been shown to correspond with significant increases in visual perceptual capacity (Fischer and Hartnegg, 2000), which could be an indication of increased phase-locking to a stable theta stimulus.

**FIGURE 5:**
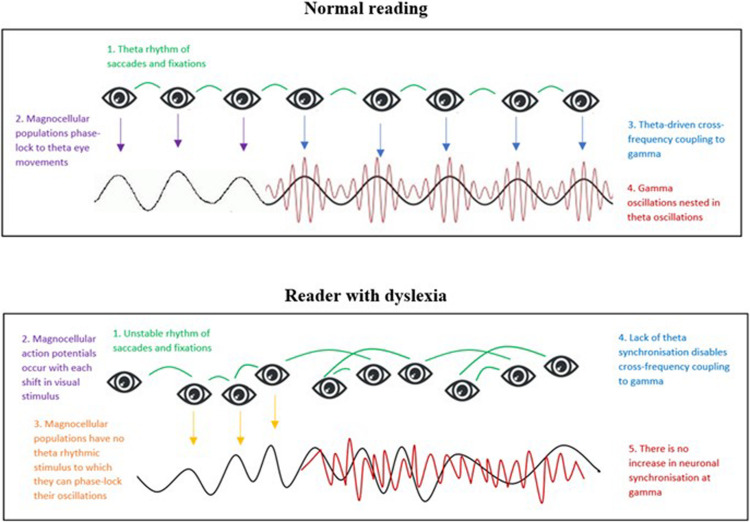
Representation of a hypothesized bottom-up model of visual temporal sampling during normal reading (Model 1). **(Top figure)**: Normal reading. 1. Eye-movements during reading occur at a theta-frequency and act as an entrainment stimulus. 2. Magnocellular oscillations in visual sensory areas phase-lock to the theta-rhythm of eye-movements, thus enabling visual coding of text. 3. Theta phase-locked oscillations drive cross-frequency oscillatory coupling to gamma. 4. Gamma oscillations become nested within entrained theta oscillations, thus enabling transfer of information to higher-order visual areas for processing of text. **(Bottom figure)**: Reader with dyslexia. 1. Dyslexic readers have erratic eye-movements during reading, generating no steady rhythm of visual stimulus shifts. 2. The action potentials of magnocells respond to each stimulus shift. 3. However, a lack of theta eye-movements means there is no stable rhythm to which magnocellular populations can entrain and this impairs coding of text. 4. Lack of theta synchronization in visual sensory areas means that cross-frequency coupling to gamma cannot occur, thus hindering communication of information along the magno-dorsal pathway 5. Lack of increased gamma synchronization in the PPC results in impaired processing of text.

However, as we described above, it is unlikely that theta entrainment alone would suffice for complex processing of text. We predict that visual temporal sampling as a bottom-up process could also drive modulation of gamma oscillations for higher-order processing of text. We also suggest that, parallel to the auditory TSF, poor theta phase-locking to eye movements in dyslexic subjects would impair effective theta-gamma coupling (refer to Figure 5).

One problem with a stand-alone bottom-up model of a visual TSF is that it does not account for the neuronal activity required to drive oculomotor activity. Attending to speech and reading a text are quite different processes. Listening to language is an exogenous attentional task, which, in comparison, is relatively passive, whereas reading involves endogenous, top-down attention and active, intentional control of eye movements. Our brain has no control over when another person speaks, necessitating rapid activation of temporal sampling of speech sounds, and bottom-up processing of sensory input. However, reading requires visual and oculomotor attention to be decisively directed to text when we choose to read. A magno-dorsal attention network has been suggested to generate top-down signals that selectively bias visual cortical activity (Gitelman et al., 1999; Hopfinger et al., 2000; Corbetta and Shulman, 2002) and has been hypothesized to be activated during reading (Vidyasagar, 1999, 2013). Anticipatory control of visual attention during reading might activate a gaze control system that actively samples text in a temporal pattern that enhances processing of text by priming the visual system for input (Rajkai et al., 2007) and controlling for information overload. Thus, we will now present a hypothesis for a visual TSF – a temporal oscillatory network where top-down attention modulates neuronal activity to sample text at a theta frequency, prior to sequential letter encoding during each fixation.

## Top-Down Processes in Oscillatory Synchronization During Reading

As discussed earlier, a cross-frequency coupling relationship between gamma and theta oscillations is well evidenced (Jensen and Colgin, 2007; Lisman and Jensen, 2013). Although this relationship is most commonly described as low-frequency oscillations modulating the amplitude of higher frequency oscillations, there is also evidence of reverse directional functional coupling. Neurons not only modulate their firing in response to changes in stimulus (bottom-up), they also modulate their firing when attention is directed to an anticipated visual task (top-down) (Itti and Koch, 2001). Synchronized gamma oscillations in higher-order processing areas predict action potentials in lower down neuronal populations, causing high-to-low frequency coupling (Volgushev et al., 1998; Tallon-Baudry and Bertrand, 1999; Di Garbo, 2008; Chelazzi et al., 2011; Bosman et al., 2012; Squire et al., 2013; Buschman and Miller, 2014; Peelen and Kastner, 2014). Top-down highlow frequency coupling has been suggested to enable task-dependent top-down neuronal communication between different brain areas (Tallon-Baudry and Bertrand, 1999; Jiang et al., 2015). In the task of attending to visual stimuli, gamma-driven theta synchronization has been suggested to enhance sensory encoding of visual environments (Itti and Koch, 2001). This mechanism may occur during reading, whereby attention modulated top-down gamma theta coupling along the magno-dorsal pathway could enable encoding of text. Indeed, increases in frontal-occipital theta and gamma synchronization have been observed immediately prior to reading target words (Molinaro et al., 2013).

**FIGURE 6:**
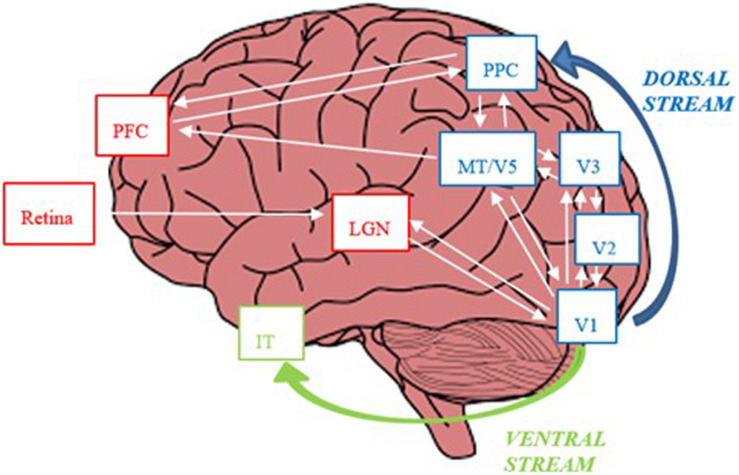
The magno-dorsal circuit. During bottom-up visual processing during reading signals from retinal magnocells are projected to magnocellular layers of the lateral geniculate nucleus (LGN). From the LGN, signals are projected from the LGN to V1 (visual cortex), where, the visual pathway diverges into the dorsal (“where”) and ventral (“what”) streams. The dorsal pathway, dominated by magnocells, is constituted by a hierarchy of cortical areas, namely, V2, V3, MT/V5 and the posterior parietal cortex (PPC). In top-down visual attention during reading, neuronal signals are sent from the PPC to MT/V5, V2, V1 and LGN. The PPC and MT/V5 also project to the pre-frontal cortex (PFC), which plays a key role in the control of eye-movements during reading. The ventral stream, proceeds to V4 and further on to the inferior temporal cortex (ITC).

**FIGURE 7:**
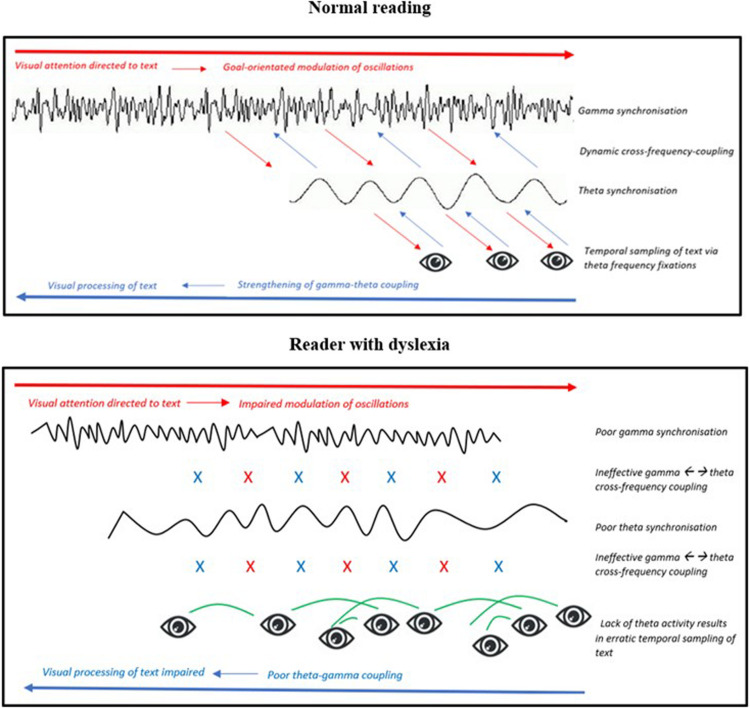
Representation of the hypothesized magno-dorsal temporal sampling framework as a top-down and bottom-up process (Model 2). (**Top figure)** Normal reading: When visual attention in the posterior parietal cortex (PPC) is directed to text it drives a top-down modulation of oscillatory activity. Oscillations in the PPC synchronize to gamma and drive high-to-low cross-frequency coupling to theta. When oscillations in visual sensory areas synchronize at a theta frequency it causes eye-movements during reading to also occur at a theta frequency. (**Bottom figure)** Reader with dyslexia: When visual attention is directed to text, oscillations in the PPC fail to synchronize effectively at a gamma frequency. This in turn hinders also the effectiveness of the PPC in modulating top-down signals required for theta-controlled eye-movements during reading. Erratic eye-movements mean visual areas lack theta-phase-locking reinforcement, thus interrupting consolidation of theta-gamma activity and hindering bottom-up information transfer.

Coupling of top-down attentional signals to lower frequencies during reading has also been suggested to contribute to word-to-word shifts of attention that occur with eye movements (Vidyasagar, 2019). This is consistent with the magno-dorsal pathway’s involvement in visual attention and oculomotor control (Nobre et al., 2000; Moore et al., 2003). Thus, an alternative hypothesis to Model 1 is that gamma theta coupling drives temporal sampling of text via theta frequency saccade-fixation movements. This is consistent with the auditory temporal sampling of theta frequency syllabic speech segments. We hypothesize in this model that theta frequency eye movements in turn reinforce theta phase-locking and propagates theta gamma coupling to complete an oscillatory circuit along the magno-dorsal pathway (see Figures 6, 7). Consistent with general properties of cortical oscillatory networks, gamma synchronization might enable neuronal functioning at the local magnocellular population level, while theta synchronization might enable long-range communication and coordination between discrete magnocellular populations along the dorsal pathway (von Stein and Sarnthein, 2000; Buzsáki and Draguhn, 2004). Furthermore, visual working memory appears to encode temporal information at a theta frequency and spatial information within gamma oscillations (Roberts et al., 2013). Thus, the low-spatial high-temporal functional characteristics of magnocells might function optimally at theta and gamma frequencies during visual processing, and particularly so, given the low-spatial high-temporal characteristics underlying eye movements during reading.

A possible source of the low, possibly theta, frequency input to early visual areas needed to parse text into chunks of letters in sync with eye movement shifts may be the claustrum. This is consistent with the recent proposal (Vidyasagar and Levichkina, 2019) of a detailed neuronal framework for a percipient suggestion by Crick and Koch (2005). In view of claustrum’s extensive anatomical connectivity with almost every cortical area, Crick and Koch (2005) suggested that its function may be to orchestrate and integrate activities across cortical areas, mediating a particular cognitive function at any instant of time. Smythies et al. (2014) have suggested that this may be done by facilitating neural synchrony between cortical areas. In providing a plausible neural basis for such a function, Vidyasagar and Levichkina (2019) suggest that claustrum’s low frequency output may serve to amplify, by cross-frequency coupling, synchronized oscillations between cortical areas that are working together at any one time to execute a cognitive function. Since claustrum is the one brain region that is connected nearly reciprocally with every cortical area, in the case of reading a text, claustral output may serve to synchronize eye movements with the coarse parsing of text and, simultaneously, boost neural synchrony in the beta/low gamma range that is needed for parsing graphemes (Vidyasagar, 2013). The model describes a framework for the fast feedforward inhibitory circuit (Bruno, 2011) implicated in the claustral model (Vidyasagar and Levichkina, 2019). This circuit may play a critical role not only in generating oscillations (Kremkow et al., 2010), but also in enhancing neural synchrony between the relevant cortical areas, pushing along the processing of graphemes at a gamma frequency and chunks of graphemes that are processed between saccades at a theta frequency. Reading requires processing of visual stimuli at multiple temporal scales (Graboi and Lisman, 2003) and if cross-frequency coupling is the mechanism that permits this, the claustrum is a good candidate to function as a central integrator that can facilitate synchrony at two temporal scales and also, by virtue of its unique connectivity, help to punctuate the stream of visual stimuli in the text, as described elsewhere (Vidyasagar and Levichkina, 2019). Though the application of the claustral hypothesis to reading is largely speculative and needs further empirical evidence, there is an intriguing fMRI study that shows strong bilateral recruitment of the claustrum by active learning of letters compared to passive viewing (Kersey and James, 2013). Imaging studies of claustrum and its functional connections in normal readers and dyslexic subjects may reveal whether claustral activity is intimately involved in reading as proposed here.

### Model 2: A Visual-Temporal Sampling Model as a Top-Down Bottom-Up Magno-Dorsal Oscillatory Circuit

A possible alternative to Model 1 is a magno-dorsal TSF where visual processing during reading involves a dynamic interplay between top-down attention-driven oscillations and bottom-up stimulus evoked oscillatory synchronization. This is consistent with evidence presented earlier of widespread bi-directional theta and gamma phase-locking during word and sentence reading (Molinaro et al., 2013; Bedo et al., 2014). In this model, theta and gamma oscillations synchronize as a mechanism for top-down predictive optimization of magno-dorsal cortical activity, as well as a bottom-up adaptive operation to enhance magno-dorsal processing during reading. It is possible that temporal sampling of text is driven by attention-modulated synchronization of higher-order magnocellular oscillations to a gamma frequency. This could drive a top-down goal-oriented modulation of magnocellular populations to synchronize at theta for the organized temporal sampling of text (see Figure 7), a process that may be orchestrated by the claustrum (Vidyasagar and Levichkina, 2019). The combined evidence of altered neuronal networks, magnocellular damage, impaired magnocellular oscillatory activity, significantly lower gamma activity in magno-dorsal processing and the lack of theta frequency eye movements during reading, suggests this oscillatory circuit may be impaired in dyslexic readers.

Given that top-down attentional signals from PPC to frontal eye fields (FEF) (Buschman and Miller, 2007) and from PPC to middle temporal visual area (MT) (Saalmann et al., 2007) have been identified, as well as top-down signals involved in visual search from the PPC to MT and then on to V1 (Vidyasagar, 1998; Brefczynski and DeYoe, 1999; McAdams and Reid, 2005), modulation of gamma oscillations during reading might arise from the “back end” of the dorsal stream network (see Figure 6). We hypothesize that this occurs in the PPC, where increased gamma activity is shown to be linked to visual attention and planned saccadic movements (Saalmann et al., 2007; Siegel et al., 2008; Van Der Werf et al., 2008; Marshall et al., 2015; Levichkina et al., 2017; Richter et al., 2017). Attention-driven gamma signals directed from the PPC to the magno-dorsal V5/MT that have been identified (Saalmann et al., 2007) may be deployed by visual attention during eye movements and visually guided movements in general (Sereno, 1992; McPeek et al., 1999; Krauzlis, 2004; Bakola et al., 2007; Laycock and Crewther, 2008; Van Der Werf et al., 2008; Ronconi et al., 2014). Signals from the PPC also project to the FEF (Tian and Lynch, 1996), where neuronal activity is thought to predicate premotor deployment of visuo-spatial attention (Vernet et al., 2014), fixations (Izawa et al., 2004, 2009), smooth visual pursuit (Bizzi, 1968; MacAvoy et al., 1991), and intentional saccadic movements (Robinson and Fuchs, 1969; Bruce and Goldberg, 1985; Luna et al., 1998; Everling and Munoz, 2000; Lachaux et al., 2006). Furthermore, modulation of LGN oscillatory activity has been found to occur 300 ms before the beginning of a saccade (Lee and Malpeli, 1998), consistent with the notion of top-down modulation of neurons even in early sensory nuclei for the control of theta frequency eye movements.

This suggests that instead of eye movements during reading initiating theta phase-locking (as would occur in Model 1), top-down oscillatory synchrony could possibly drive the theta frequency oculomotor mechanics of attention, significantly enhanced by the claustrum as explained earlier. Anticipatory control of visual attention would enhance processing of attended stimuli by biasing the visual cortex before stimulus onset, priming the visual system for a temporal pattern of visual input that is a straightforward consequence of gaze control systems (Rajkai et al., 2007; Bosman et al., 2012). Synchronized gamma signals might enable the spatio-temporal filtering of text by instigating theta oscillatory synchronization that controls eye movements, in a manner inverse of bottom-up signals in the auditory TSF. In other words, eye movements during reading could be driven by top-down goal-orientated gammatheta signal along the magno-dorsal-dorsal pathway. Applying Model 2 to dyslexia, ineffective gamma production could result in a theta magno-dorsal oscillatory deficit, resulting in erratic eye movements and thus poor temporal sampling of text by magno-dorsal-cells. Poor temporal filtering of text would bombard the magno-dorsal visual system with an overload of disordered information, affecting the encoding of magno-specific text characteristics as well as affecting the gating of graphemic information into the ventral stream for identification of letters and their temporal sequence. Thus, the full story of visual temporal sampling in dyslexia may in fact be a synthesis of a bottom-up mechanism (mirroring the auditory TSF) and a top-down mechanism (driven by the PPC and boosted and sequenced by the claustrum), creating a magno-dorsal oscillatory circuit which is impaired in dyslexia (refer to Figure 7).

### An Oscillatory Circuit

In an oscillatory “loop,” bottom-up entrained theta oscillations might strengthen neuronal communication during reading via reciprocal frequency coupling to gamma – in other words, top-down modulation of oscillations (as in Model 2) and bottom-up phase-locking (as in Model 1) might occur in concert to enable reading. Given that retinal magnocellular gamma activity is evidenced in the FD illusion (Pammer and Wheatley, 2001; Gori et al., 2014), as well as findings of gamma activity in the LGN (Neuenschwander and Singer, 1996; Castelo-Branco et al., 1998), it is possible that simultaneous theta and gamma activity occur along the entire magno-dorsal pathway, such that each frequency is nested within one another and modulating each other (Penttonen and Buzsáki, 2003; Le Van Quyen, 2011; Vidyasagar and Levichkina, 2019). Early support of such a circuit is in the identification of a rapid oscillatory loop between V1 and the LGN, where top-down signals and bottom-up signals influence each other, strengthening communication between the two areas (Briggs and Usrey, 2007).

The functional purpose of such a circuit could be three-fold. Firstly, it would enable the rapid communication between brain areas that is needed during reading. While gamma oscillations might enable operations at the local magnocellular population level (Lisman and Buzsaki, 2008), theta oscillations might temporally organize these population-level operations across different brain regions along the dorsal pathway (Lisman and Buzsaki, 2008). Secondly, reading graphemes (letters) in text occurs at a low gamma frequency. The average typical reading speed is 303 words per minute (Rubin and Turano, 1992), equating to approximately 23 graphemes per second – in other words, 23 Hz (Vidyasagar, 2013). This is also consistent with the temporal frequency of phonemes in spoken language (Vidyasagar, 2013). Theta–gamma synchronization in sensory visual areas might act as a “neural code” to enable discrimination and temporal ordering of sequential stimuli (Lisman and Buzsaki, 2008). While theta frequency eye movements “funnel” stimulus to control for information overload, simultaneous gamma oscillations might encode single letters, prior to processing letter-sequences (i.e., words) (Vidyasagar, 2013). Thirdly, as discussed earlier, gamma signals not only increase with attention *before* visual stimulus presentation, but also after stimulus onset, enabling detailed processing of bottom-up signals from external stimuli (Tallon-Baudry and Bertrand, 1999; Fries et al., 2002; Fell et al., 2003; Saalmann et al., 2007), consistent with concepts of top-down gating of incoming sensory stimuli (von der Malsburg, 1981; von der Malsburg and Schneider, 1986; Treisman, 1988; Vidyasagar, 1999). Thus, pre-stimulus top-down gamma synchronization might be enhanced by eye movement evoked phase-resetting of oscillations in visual sensory areas (Tallon-Baudry and Bertrand, 1999; Fries et al., 2001; Fell et al., 2003) and by the claustral circuit via theta-gamma coupling (Vidyasagar and Levichkina, 2019). In this way, the amalgamation and transfer of top-down attention and bottom-up information processing may be enabled by a two-way theta gamma coupling circuit (von Stein and Sarnthein, 2000; Demiralp et al., 2007; Holz et al., 2010; Bastos et al., 2012; Friese et al., 2013; Wang et al., 2014). Conversely in dyslexia, unstable theta activity in oculomotor control could destabilize theta gamma oscillatory coupling and affect bottom-up transfer of textual information (see Table 2 for a summary of some of the evidence supporting a top-down and bottom-up magno-dorsal oscillatory circuit).

**TABLE 2:** Some of the evidence of theta and gamma oscillatory processes along the magno-dorsal pathway during visual processing.

Study	Experiment details	Results
Bottom-up oscillatory signals		

Ruff et al., 2006	Conducted transcranial magnetic stimulation (TMS) over human FEF and compared trials with and without visual stimuli. Measured changes in fMRI activity	TMS modulation of oscillations over the FEF increased fMRI activity in V1, V2 and V3, and increased the perception of peripheral visual stimuli
Frien et al., 1994	Single-cell recordings taken in V1 and V2 of an awake monkey during fixations on visual stimuli	Stimulus induced fixations occurred with phase-locking of oscillations at gamma (50–90 Hz) between V1 and V2
Bastos et al., 2014	Local field potential recordings in monkeys in LGN and V1 in response to visual stimuli	Bottom-up LGN – V1 phase synchronization in response to visual stimuli was positively correlated with synchronization at 2–10 Hz Gamma synchronization (44–52 Hz) was observed in V1 but not LGN in response to visual stimuli
Demiralp et al., 2007	EEG recording from participants whilst completing a visual perception and short-term memory task	Stimulus induced increase in gamma oscillations, correlated with the phase of theta oscillations
McClurkin et al., 1991	Single-cell recordings from LGN in monkeys during presentation of visual stimuli that varied in shape and complexity	Temporal patterns of response of neuronal firing in LGN corresponded to changes in shape of visual stimuli
Reich et al., 2001	Single-cell recordings from V1 in monkeys during sinusoidal grating visual stimulus	Observed temporal coding in V1 in response to contrast information in stimulus and timing of neuronal spikes varied as the spatial frequency varied. Suggested that temporal coding in V1 enables the visual pathway to distinguish among stimuli that evoke similar neuronal firing rates
Fries et al., 2002	Single-cell recordings in visual cortex of cats while presenting contrasting visual stimuli with a temporal offset	Found increased gamma synchronization in visual cortex in response to stimulus changes
Lutzenberger et al., 1995	EEG recordings from 12 human subjects while viewing a changing visual pattern	Increased gamma (40 Hz) activity when changes in visual stimulus appeared in a regular temporal pattern
Izawa et al., 2009	Single-cell recordings in FEFs of 2 monkeys during a visual attention task	Neurons in the FEF increased their firing rate at the start of a fixation and continued discharging during fixation
Kreiter and Singer, 1996	Single-cell and multi-unit recordings taken in the V5/MT area of 2 monkeys while viewing a moving light visual stimulus	MT/V5 oscillations synchronized in response to light stimulus

**Top-down oscillatory signals**		

Van Der Werf et al., 2008	MEG data collected during a visual stimulus designed to direct saccades toward and away from flashing stimulus	Increased gamma synchronization in PPC found just before initiation of saccades toward the stimulus, suggesting PPC gamma involvement in controlling and planning saccades
Bakola et al., 2007	Injected C-deoxyglucose was used to trace neuronal activity in 6 monkeys during a visually-guided saccade task, and a memory saccade task	Top-down signals deployed from MT/V5-foveal (which represents central vision) during visually-guided saccades and memory-guided saccades
Izawa et al., 2004	Micro-electrode stimulation conduced in the FEFs of 2 monkeys during a visual-behavioral task with eye-tracking	Top-down oscillatory projections from FEF increased with stimulation, associated with motor control of fixations and saccades.
Bruce et al., 1985	Performed micro-stimulation in FEF neurons of monkeys and compared neuronal firing to eye-movements	Top-down oscillatory projections from FEF caused saccadic movements
Everling and Munoz, 2000	Single-cell recordings from FEFs in 2 monkeys during saccade task	Top-down oscillatory projections from FEF directly associated with attention-driven saccadic movements
Lachaux et al., 2006	Direct intracranial EEG recordings from FEFs in 3 humans to investigate time-course of oscillatory changes during saccadic movements	FEF activity >60 Hz associated with preparation and production of saccades
Holz et al., 2010	EEG recordings while participants performed a visuo-spatial short-term memory task	Phase-matched theta and gamma activity occurred during task
Saalmann et al., 2007	Single cell and local field potentials recorded simultaneously from areas LIP and MT in macaques	Top-down signals from LIP to MT, with coherence in the gamma range, led to attentional enhancement of MT neuronal activity

**Top-down bottom-up oscillatory circuit**		

Tallon-Baudry et al., 1997	EEG recordings from 13 human participants, and time-frequency analysis used to assess oscillatory response to visual search task	Gamma (35–38 Hz) oscillations phase-locked to temporal rate of changes in stimuli. Gamma amplitude also increased ∼280 ms post stimulus onset. Supports gamma synchronization occurs with top-down visual search and bottom-up feature-binding
Tallon-Baudry et al., 1996	EEG recordings from 8 human participants, and time-frequency analysis used to assess oscillatory response to changes in visual stimulus	Early increased gamma (40 Hz) synchronization which did not vary with changes in stimulus (possibly indicating top-down gamma increasing with visual attention). A second increase in gamma (40 Hz) at ∼280 ms post stimulus onset that required more complex feature binding
Bastos et al., 2014	Macaque study. Recorded simultaneous local field potentials in retinotopically aligned regions in the LGN and V1. Presented drifting grafting visual stimuli	Increase in visually evoked gamma power (30–100 Hz) in V1 and synchronized oscillations at 15–30 Hz with top-down interactions from V1 to LGN and increase in oscillations at 8–14 Hz with bottom-up interactions from LGN to V1
Rajkai et al., 2007	Studied visual fixations, EEG and intracranial recordings in V1 in macaques. Analyzed stimulus vs. fixation related neuronal activity.	Excitation commenced at fixation onset and continued for approx. 200 ms. This time frame brackets the arrival time of retinal inputs to V1 (bottom-up). Found significant phase concentration at 3–8 Hz, from 300 ms pre-fixation to 77.5 ms post fix. (top-down), but no sig. effects in other bands
Briggs and Usrey, 2007	Single-cell recordings from V1 and LGN while electrically stimulating the LGN of 7 monkeys as they observed visual stimulus	Stimulation of LGN caused rapid bottom-up excitation in V1, followed by rapid top-down projections back to the LGN
Bosman et al., 2012	Single-cell recordings from V1 and V4 in macaques in paradigm that led attention to select one of two stimuli	Attention causes selective synchronization of bottom-up signals from V1 to V4

## Implications for Future Research

### Testing the Hypothesis of a Visual TSF

Much of the evidence for top-down and bottom-up oscillatory activity during visual processing so far comes from electrophysiological studies on monkeys. However, exploring the possibility of a magno-dorsal correlate to the auditory TSF would also require human studies using normal readers and dyslexic readers. One of the strengths of the visual TSF models we have presented is that they each generate a number of testable hypotheses. Identifying whether a magno-dorsal visual TSF exists in reading and is impaired in dyslexia could be investigated experimentally with functional imaging (for oscillatory activity and anatomical locations of that activity), eye tracking, transcranial stimulation and behavioral entrainment. Magnocellular-isolating stimuli have typically been used in studies to investigate differences between the two populations, however it will also be important to conduct experiments using reading tasks which require coding and cognitive processing of written language that cannot be fully replicated in other visual tasks. Preliminary aims of investigations would likely be to (1) identify if oscillatory synchronization at theta and gamma frequencies along the magno-dorsal pathway does indeed occur during reading; (2) identify whether magno-dorsal oscillatory synchronization occurs only in a bottom-up manner (i.e., do theta eye movements entrain theta oscillations and then drive gamma synchronization?) or in a top-down-bottom-up circuit (i.e., does gamma oscillatory activity initiate in PPC when a person starts reading? And is this followed by increased theta in sensory areas?) (3) is there a difference between dyslexic and non-dyslexic readers in the functioning of magno-dorsal oscillatory synchronization? What is the specific cognitive-behavioral effect of this difference? (4) Does the claustrum play a critical role in boosting the synchrony between cortical areas? and (5) Does claustral input to visual areas operate in the theta frequency apparent during reading, especially involving the areas classically known to be activated during reading, such as the parietal cortex and the visual word form area (VWFA)?

Isolating the specific phase-locked oscillatory activity between brain areas in visual processing using single cell recordings is one possibility. However, given that single-cell recordings are highly unlikely to involve human participants (these are typically conducted using monkeys) this method would be of less use in investigating magno-dorsal activity during normal and dyslexic reading. However, local field potentials from electrocorticogram (ECOG) from surface recordings in patients implanted with electrode arrays typically to investigate epileptogenic foci is a possibility for testing our models, if these areas happen to involve parietal and early visual areas.

Bottom-up oscillatory entrainment could be investigated by examining the relationship between individuals’ eye movements during reading and theta and gamma activity. The use of eye tracking technology combined with neural imaging (ideally fMRI and MEG) that is time-locked to the eye tracker would enable oscillatory patterns to be examined in direct relation to fixations and saccades during reading. Given that individuals vary in both reading speed and resting alpha activity, it would be expected that their individual eye movement frequency would directly reflect their individual theta waves (between 3 and 7 Hz) if there is a direct association between the two (i.e., average eye movement frequency within the theta window would be same theta frequency as magnocellular oscillatory activity, with significant coherence between their phases). It would also be expected that along with erratic eye movements, dyslexic readers would demonstrate significantly lower theta activity. Eye tracking and imaging would similarly enable investigation of gamma synchronization during reading. Time-sequencing of neural activity would be important in examining the order of oscillatory changes in specific brain locations along the magno-dorsal pathway and may further the identification of top-down or bottom-up activity during reading, and possible oscillatory impairments in dyslexic readers.

One non-invasive technique that has been suggested as being effective in influencing cognitive functions and that can be used to test the validity of the visual TSF is transcranial electrical stimulation (Fertonani and Miniussi, 2018). Admittedly, one still needs to be cautious of the efficacy of the method (see Horvath et al., 2016; Vöröslakos et al., 2018; Vidyasagar, 2019). The technique involves applying electrical stimulation on the scalp, either as direct current (tDCS) or alternating current (tACS). If the technique is proven to be of some effect, tACS can be used along with imaging and eye tracking studies to examine the direct relationship between synchronized oscillations and eye movements. The frequency of the alternating current can be adjusted according to experimental aims, with the conducting electrode placed on the scalp corresponding to a specific brain location of interest. When set to a specific frequency, the tACS electrical impulses are thought to stimulate neuronal firing in the underlying brain area. In this way, “enforced” oscillatory entrainment may be stimulated within neural populations. The effects of tACS can be measured both behaviorally and with imaging. Conducting tACS at a theta frequency over an area such as the FEF or MT/V5, combined with eye tracking and imaging, could be of interest in examining any top-down effects on eye movements. Furthermore, if bottom-up theta → gamma coupling occurs with theta frequency eye movements, it would be expected that an increase in gamma activity might be seen in the PPC. Alternatively, gamma frequency tACS could be applied over the PPC during reading. If a top-down gamma → theta network is in play, we would expect an increase in theta activity in sensory areas. If continuous theta ↔ gamma coupling occurs along the magno-dorsal pathway, we might expect to see an increase in gamma activity as well as theta activity in areas such as area V1 and perhaps even the LGN when gamma tACS is applied over the PPC. It would be important to examine any cognitive effects of tACS entrainment and we would expect that it would increase the effectiveness of visual processing during reading – particularly in readers with dyslexia. This raises the possibility of therapeutic entrainment studies in dyslexic readers. However, given the controversy surrounding the effectiveness of transcranial electrical stimulation (reviewed in Vidyasagar, 2019), much work is needed to investigate the usefulness of this approach for correcting any temporal sampling deficit in dyslexia.

### Therapeutic Neuronal Entrainment in Dyslexia

Although in its infancy, behavioral brainwave entrainment as a remedial tool is gathering evidence. Whereas tACS is not endorsed for use in children due to unknown long-term adverse effects, behavioral entrainment is safe and non-invasive. Given that dyslexia is a childhood developmental disorder, a neurological therapy should be child-friendly and could be of great use in targeting the brain-basis of dyslexia. The effectiveness of visual entrainment was demonstrated by Herrmann (2001) who used custom-made goggles that emitted a rapid flashing light at various frequencies (between 1 and 100 Hz). Oscillatory activity in response to the light significantly increased corresponding to the frequencies of 10, 20, 40 and 80 Hz. This light flicker entrainment mechanism has been since demonstrated in other imaging studies (de Graaf et al., 2013; Spaak et al., 2014).

Positive effects of oscillatory entrainment have been demonstrated in attentional disorders (Weaver et al., 2012), epilepsy (Auvichayapat et al., 2013), motor control (Valle et al., 2007), depression (Bloch et al., 2008), Tourette’s syndrome (Kwon et al., 2011), communication and behavioral difficulties related to Autism Spectrum Disorder (Baruth et al., 2010), and schizophrenia (Mondino et al., 2015a, b). Applied to dyslexia, visual theta and/or gamma entrainment might optimize the condition of oscillatory activity in the magno-dorsal pathway. Combining reading exercises with behavioral brainwave entrainment in dyslexic readers could be a way of priming neuronal networks in the brain to be optimally receptive to reading.

There is evidence for significant improvements in reading scores (speed and comprehension), selective attention, both visual and auditory working memory, and phonological processing in dyslexics from discrimination training in visual motion perception when conducted relative to a stationary background frame of reference, thereby being an effective training stimulus to improve magno-parvo integration deficits at both early and higher levels of motion processing (Lawton, 2016; Lawton and Shelley-Tremblay, 2017). The background frame of reference entrains motion discrimination, since it repeats at the same periodicity (harmonically-related) as the test pattern moving left or right (Lawton, 1985). Increasing the ease of magno-parvo integration by oscillatory entrainment of motion discrimination facilitates figure/ground discrimination within a wider window of focused attention, improving reading skills by strengthening coupled theta/gamma and alpha/gamma activity (Lawton, 2016; Lawton and Shelley-Tremblay, 2017).

In addition, the possibility of a multi-modal auditory and visual temporal sampling deficit in dyslexia opens the door for remedial cross-domain behavioral entrainment. For example, auditory entrainment (typically a rhythmic pulse) has been shown to improve temporal allocation of visual attention (Ronconi et al., 2016a) as well as conscious visual processing (Ronconi et al., 2016b). Furthermore, cross-domain motor-to-auditory entrainment can be seen with tactile behavioral tasks (Plöchl et al., 2016). Along with entrainment in the visual and auditory domains, behavioral entrainment that also incorporates the motor-kinaesthetic domain might optimally enhance phase-locking required for reading. Remarkably, studies have demonstrated a remedial effect of playing action video games (AVGs) – which by design incorporate rapid and repetitive auditory, visual and motor stimulation – in children with dyslexia. After two weeks of daily AVG playing, children with dyslexia have demonstrated significant improvements in attentional and reading abilities (Franceschini et al., 2013), reading speed – without any cost in reading accuracy (Franceschini et al., 2013), word recognition, visuo-spatial attention, visual-to-auditory attention shifting (Franceschini et al., 2017), phonological decoding and short-term phonological memory (Franceschini et al., 2017; Franceschini and Bertoni, 2019). Furthermore, AVG players show improvements in dorsal processing, including motion discrimination (Hutchinson and Stocks, 2013) and contrast sensitivity (Li et al., 2009). Given the high-temporal low-spatial visual and auditory characteristics of AVGs, as well as the high-temporal motor-kinaesthetic response necessitated when playing, it is possible that AVGs stimulate oscillations across sensory domains in multi-modal magnocellular pathway.

### Could Magnocellular Oscillatory Impairment Underlie the Auditory and Motor Deficits in Dyslexia?

Magnocells are not unique to the visual system, and communication between the visual and auditory domains, which is essential for rapid visuo-phonological mapping during reading, may occur via magnocellular oscillations. Magnocellular layers are also found in the medial geniculate body (MGB) of the auditory thalamus (Wu et al., 2015). Action potentials of MGB magnocells are tone-evoked and have the capacity for rapid development of discharge plasticity in response to auditory signals, enabling the temporal sequencing of transient sounds (Gerren and Weinberger, 1983; Wu et al., 2015). Furthermore, synaptic communication between sensory domains relies heavily on temporal sampling (Wu et al., 2015), and there is significant evidence that oscillatory entrainment in response to behavioral stimuli occurs simultaneously in the auditory and visual domains (Cooper, 1974; Calvert et al., 1997; Hasselmo, 2005; Meltzer et al., 2007; Jaušovec and Jaušovec, 2014). As well as auditory input, the magnocellular MGB receives visual afferents, contributing to a cross-sensory “conditional circuitry” (Gerren and Weinberger, 1983; Wu et al., 2015). Conversely, the PPC receives auditory as well as dorsal stream visual input. Furthermore, cross-modal oscillatory communication appears to occur with gamma synchronization (Fell et al., 2003), consistent with evidence of gamma involvement in reading and dyslexia (Lehongre et al., 2011). Thus, as well as enabling cognitive processing during reading, gamma oscillations might be integral to neuronal interaction between the visual and auditory pathways.

Beyond auditory and visual processing difficulties, children with dyslexia often display difficulties with fine motor skills (Nicolson and Fawcett, 1994; Fawcett and Nicolson, 1995), balance (Nicolson and Fawcett, 1994) and articulation (which requires verbal motor-kinaesthetic skills) (Fawcett and Nicolson, 1995). As well as oculomotor control during reading, magnocells are involved in generalized motor signals (Kennedy, 1987; Robinson et al., 1987; Van Kan and McCurdy, 2001), and it has been suggested that the motor deficits in dyslexia are also the result of a damaged magnocellular network (Stein and Talcott, 1999; Stein, 2019).

A possible region of interest for future investigations into a multi-modal magnocellular entrainment deficit could be the posterior parietal cortex. The PPC is in one way a “hub” for cross-domain neuronal interactions – it receives visual, auditory and somatosensory input, as well as processing premotor signals. Differences between dyslexic and non-dyslexic readers in processing temporal information in the magnocellular layers of the PPC would support the hypothesis of a multimodal magnocellular oscillatory network.

## Conclusion

Like the temporal sampling of syllabic speech-sounds hypothesized in the auditory TSF, visual processing of text requires the rapid serial sampling of text. This occurs via sequential fixations that enable effective coordination of the cognitive visual process required to read, e.g., visual attention, feature binding and spatial coding (Cornelissen and Hansen, 1998; Rayner, 1998; Vidyasagar, 1999, 2013; Vidyasagar and Pammer, 1999, 2010; Pammer et al., 2004a, b; Facoetti et al., 2006, 2010; Sireteanu et al., 2008; Pammer, 2014; Moore and Schwitzgebel, 2018). Given that text is a static stimulus, temporal coding occurs via rapid processing of visual information that is gathered during saccades and fixations. It has been highlighted that during reading, these fixations occur every 200–300 ms (Rayner, 1998) – a theta frequency (Pammer, 2014). Here we propose that this is reflective of theta synchronized oscillatory activity in lower-order areas of the visual pathway, mirroring the auditory domain’s theta synchronization in response to speech sounds. However, dyslexic readers demonstrate significant differences in oculomotor control during reading, with no regular rhythm of saccades and fixations. We suggest that this signifies a deficit in visual temporal sampling of text and synchronization of oscillatory populations – specifically magnocellular populations in the dorsal stream. Given the evidence for differences in gamma synchronization between typical and dyslexic readers in higher order dorsal stream areas, along with evidence for a functional cross-frequency coupling relationship between theta and gamma oscillations, we suggest that theta and gamma oscillations couple along the dorsal stream to enable coding of text.

We have presented two alternate hypotheses for a magno-dorsal theta-gamma TSF. Model 1 is a bottom-up neuronal entrainment that directly parallels temporal sampling of spoken language in the auditory domain. In this model, we hypothesize that theta frequency eye movements during reading might entrain theta phase-locking in magnocellular populations, initiating theta–gamma coupling along the magno-dorsal pathway for the higher-order processing of text. Conversely, dyslexic readers who have unstable eye movements during reading might have no stable theta stimulus to entrain to, resulting in impaired theta phase-locking and a theta-gamma coupling deficit. The alternative hypothesis, Model 2, is a dynamic oscillatory network where theta and gamma oscillations interact in a top-down-bottom-up neuronal circuit. This visual TSF involves goal orientated “top-down” gamma signals from higher-order visual processing which couple with theta oscillatory activity in lower-order areas. Temporal sampling of text at a theta frequency may bolster theta oscillatory synchronization and reinforce coupling to gamma as visual input is fed back along the dorsal and ventral streams for higher-order processing.

The unique characteristics of magnocells suggest magnocellular oscillations play a key role in spatio-temporal capture and coding of text. Thus, a visual neuronal network which is hindered due to magnocellular anomalies in dyslexia may not only account for difficulties in low-spatial/high-temporal frequency processing, but also be the theoretical link between the TSF and magno-dorsal deficit hypotheses of dyslexia. Future testing of the presented hypotheses of a magno-dorsal TSF may begin to unite a number of disparate areas of visual processing and dyslexia to provide a cohesive, neurophysiological model of dyslexia.

## Author Contributions

KA, KP, and TV contributed to the concepts and hypotheses, manuscript revision, and read and approved the submitted version. KA wrote the first draft of the manuscript.

## Conflict of Interest

The authors declare that the research was conducted in the absence of any commercial or financial relationships that could be construed as a potential conflict of interest.

## Footnotes

1 For convenience, from this point on, we will use the term “magno-dorsal” as we recognize that it is difficult to disentangle impairments in the cellular makeup (magno) of the dorsal pathway from functioning of the pathway itself.
